# Bioavailability of Anthocyanins: Whole Foods versus Extracts

**DOI:** 10.3390/nu16101403

**Published:** 2024-05-07

**Authors:** Ravish Kumkum, Kathryn Aston-Mourney, Bryony A. McNeill, Damián Hernández, Leni R. Rivera

**Affiliations:** Institute for Innovation in Physical and Mental Health and Clinical Translation (IMPACT), Deakin University, Geelong 3220, Australia; k.ravish@deakin.edu.au (R.K.); k.astonmourney@deakin.edu.au (K.A.-M.); bryony.mcneill@deakin.edu.au (B.A.M.); d.hernandez@deakin.edu.au (D.H.)

**Keywords:** anthocyanins, bioavailability, nutraceuticals, dietary supplements, bioaccessibility, bioactivity, whole foods, anthocyanin extracts

## Abstract

Anthocyanins have gained significant popularity in recent years for their diverse health benefits, yet their limited bioavailability poses a challenge. To address this concern, technologies have emerged to enhance anthocyanin concentration, often isolating these compounds from other food constituents. However, the extent to which isolated anthocyanins confer health benefits compared to their whole-food counterparts remains unclear. This review explores the current literature on anthocyanin bioavailability and metabolism in the body, with a focus on comparing bioavailability when consumed as extracts versus whole foods rich in anthocyanins, drawing from in vitro, in vivo, and human clinical studies. While direct comparisons between anthocyanin bioavailability in whole foods versus isolates are scarce, prevailing evidence favours whole-food consumption over anthocyanin extracts. Further clinical investigations, preferably with direct comparisons, are needed to validate these findings and elucidate the nuanced interplay between anthocyanins and food matrices, informing future research directions and practical recommendations.

## 1. Introduction

In the quest for optimal health and longevity, phytonutrients have emerged as remarkable bioactive compounds found abundantly in various plant-based foods. Anthocyanins, a major class of flavonoids and a phenolic phytonutrient, have gained significant attention for their beneficial health-promoting properties, including anti-inflammatory [1,2,3], antioxidant [4,5], anticancer [6,7,8], immunomodulatory [9,10], antimicrobial [11], antiaging [12], cardioprotective [13], hypoglycaemic, and hypolipemic properties [14]. With increasing evidence supporting their health benefits, the 21st century has witnessed a resurgence in research studies focused on anthocyanins [15]. As a result, anthocyanins have attracted significant attention from the food, medicine, and therapeutic industries. Their applications extend from being used traditionally as food colourants to functional foods. However, due to their poor stability and solubility properties, their bioavailability is often limited. Moreover, the concentration of these bioactive phytonutrients can vary significantly due to a range of factors, such as differences in plant species and cultivars, seasonal and environmental factors, agricultural practices, food processing techniques, and storage methods [16]. As a result, various food processing methods and techniques have been specifically employed in an attempt to preserve or enhance these bioactive compounds, thereby maintaining their stability and bioactivity [17]. In addition to the focus on enhancing the anthocyanin availability in fresh foods, the incorporation of these compounds in the form of functional foods or dietary supplements such as tablets, powders, capsules, and food products has become widespread. Since the advent of advanced chromatography systems to extract and purify substantial amounts of specific bioactive compounds from complex food matrices, a significant concern has arisen regarding their bioefficacy, with a special focus on the potential health benefits of consuming isolated forms compared to whole food sources.

Isolated phytonutrients are often marketed as dietary supplements, which are also referred to as nutraceuticals, a term that combines the features of “nutrition” and “pharmaceuticals”. The nutraceuticals market has been growing dramatically in recent years, particularly following the COVID outbreak, and the anticipated growth from 2023 to 2030 is projected to be at a compounded annual growth rate of 9.4% globally [18]. Anthocyanin supplements have gained a substantial portion of the current nutraceutical market [19]. Supplements, or pure forms of phytonutrients, are consumed for various reasons, such as convenience, absence of sugar content, limited access to certain foods, dislike for certain foods, and persuasive marketing strategies. However, with this kind of shift to supplements over whole foods, individuals may miss out on benefits due to ‘food synergy’, which represents the overall effect of the food resulting from the interaction of multiple nutrients present in the food [20]. This is because studies have shown that the food matrix, referring to the composition and structure of the food itself, plays an important role and significantly affects bioavailability. While numerous studies have been conducted to analyse the bioavailability of pure forms of phytonutrients and whole foods separately, it can be challenging to compare their effects and draw conclusive findings due to variations in doses, inter-laboratory differences, the use of different methodologies, and the lack of standardised protocols. There is a notable lack of studies that directly compare the efficacy and bioavailability of isolated anthocyanin with whole foods rich in anthocyanins. Therefore, in light of the increasing interest from both the food and pharmaceutical industries in isolating bioactive compounds from whole foods and promoting them as dietary supplements or nutraceuticals, it has become imperative to investigate the effectiveness of these isolated compounds in comparison to their natural forms. This review aims to address this research gap by conducting a literature review on the bioavailability of anthocyanins based on in vitro, in vivo, and human studies with an attempt to compare their efficacy when consumed in isolated form versus whole foods rich in anthocyanins.

## 2. Anthocyanins: Sources, Types, and Structure

The term anthocyanin originates from the Greek words “anthos”, meaning “flower”, and “kyáneos”, meaning “blue” [15]. Anthocyanins are phytonutrients that belong to the flavonoid class under polyphenols and are water-soluble pigments widely distributed in nature responsible for the purple, blue, and red hues in various plant tissues [21]. They predominantly occur in the outer skin/cell layers of a variety of fruits and vegetables, as well as some grains, roots, and tubers [22].

Anthocyanins are commonly found in plant structures, including leaves, fruits, stems, and flowers [23]. Some examples of fruits that are rich in anthocyanins include blackcurrants, cranberries, raspberries, strawberries, blueberries, bilberries, red and black grapes, and plums. Vegetable sources include red-coloured cabbage, onions, and radishes, and purple-coloured eggplant, cauliflower, and corn. Even legumes and grains such as black-coloured beans, soy, and rice are some prominent dietary sources of anthocyanins [24]. These natural food sources of anthocyanins contribute to both visual appeal and various health benefits upon consumption.

**Figure 1:**
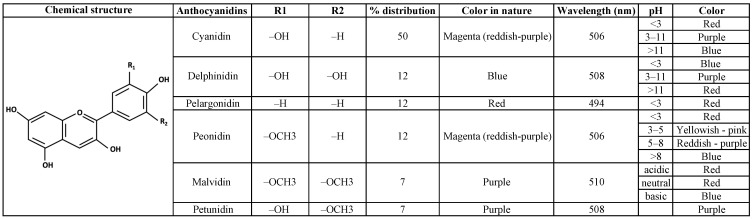
Chemical structure, percentage distribution, and properties of six anthocyanidins.

To date, over 650 different anthocyanin compounds have been identified in plants, of which 90% are represented by 6 types of anthocyanidins: cyanidin, delphinidin, pelargonidin, petunidin, peonidin, and malvidin [21] (Figure 1). In nature, cyanidin appears as a red-purple or magenta pigment and is predominantly found in berries and vegetables such as purple corn and red sweet potatoes. Similarly, delphinidin appears as a purple or a blue-reddish pigment, giving blue colour to flowers and berries. The pigment pelargonidin is present freely in nature as a red pigment in fruits but gives an orange hue in flowers. Petunidin appears as a dark red or purple pigment and is abundant in blackcurrants and purple flowers. Peonidin appears as a magenta pigment and is found in grapes, berries, and red wines. Lastly, malvidin, a purple pigment, is notably found in blue-coloured flowers and is a major component of red wines [21]. Other less commonly encountered anthocyanidins include apigeninidin, aurantinidin, europinidin, and rosinidin. Each of these compounds exhibits a specific colour in nature and is susceptible to alterations due to factors such as temperature, light, pH, oxygen, copigmentation, enzymes, water activity, sugar content, other food constituents, and food processing techniques.

Anthocyanins are derived from the flavylium (2-phenyl benzopyrylium) ion, consisting of two benzoyl rings separated by a heterocyclic ring (Figure 1). The number and positions of methyl and hydroxyl groups present as substituents in this flavylium structure give rise to anthocyanidins, the precursors of anthocyanins. The addition of a sugar moiety or a glucoside group to anthocyanidins forms anthocyanins. The presence of glucose in the structure makes anthocyanins more stable, and they are more abundantly found in nature compared to their aglycone counterpart, anthocyanidin. The most common sugars present that form the α or β linkages are rutinose, glucose, galactose, arabinose, rhamnose, and xylose. All forms of anthocyanins are almost exclusively glycosylated, except for 3-deoxyanthocyanin [25]. The number and positions of sugars, hydroxyl groups, and the presence of aliphatic or aromatic acids attached to these groups may all contribute to many derivatives of anthocyanins. Glycosylation of hydroxyl groups is often observed at the C3 position, but derivatives with glycosylation at the 3,5 and 3,7 positions, as well as replacements at 3′ and 5′, have also been identified [26]. Further, acylation of sugar residues occurs by organic acids. Some common acylating agents include cinnamic acid derivatives, including p-coumaric, caffeic, sinapic, and ferulic acids, in addition to various aliphatic acids, including malic, acetic, malonic, succinic, and oxalic acids [27]. As a result, several chemical combinations of anthocyanidins exist because of glycosylation and acylation by a variety of sugars and acids at various locations. In addition to this, anthocyanins can also interact with each other, which changes the colour and structural balance [28]. The specific modulating effects or functions of anthocyanins can be assessed by the number of hydroxyl groups and the presence of a sugar moiety [29].

Considering the low stability and sensitivity of anthocyanins to various factors, as mentioned above, several measures and techniques have been applied to enhance the stability as well as bioavailability of anthocyanins [30]. Some of them include copigmentation techniques using phenolic acids such as hydroxybenzoic acids or hydroxycinnamic acids [31], use of yeast mannoproteins at higher pH (7) [32], inclusion of suitable metal ions such as calcium ions [33], optimising food matrices using protein-binding approaches (55), and encapsulation techniques within the drug delivery system, including microencapsulation, liposomes, and nanoparticles [34,35,36], some of which have demonstrated promising results in terms of digestion stability and bioavailability.

## 3. Bioavailability: Definition and Study Designs

The concept of bioavailability initially emerged in pharmacology and is defined by the U.S. Food and Drug Administration as “the rate and extent to which the active ingredient or active moiety is absorbed from a drug product and becomes available at the site of drug action” [37]. Bioavailability examines four important processes/stages: Liberation, Absorption, Distribution, Metabolism, and Elimination (generally known as LADME) [38,39,40]. A similar concept is applied in the context of nutrition, where micronutrients and bioactive compounds are studied to understand their ability to be released by the food matrix (food structure or composition), absorbed within the body (including the bloodstream, small intestine, tissues, and organs), reach the colon, and ultimately be excreted [41]. The term bioavailability has been given various definitions by researchers; nonetheless, for phytonutrients in particular, these processes are studied in two stages, namely, (a) bioaccessibility, which is the ability of a bioactive compound to be able to withstand the digestion process and reach the colon, and (b) bioactivity, which is defined as the ability of a bioactive compound to exert a beneficial physiological effect [40].

Phytonutrients exhibit diverse chemical structures and properties, leading to variations in their bioavailability. Therefore, assessing the bioavailability of phytonutrients is crucial for understanding their potential health benefits and optimising their dietary intake. Various methods are being employed to assess phytonutrient bioavailability, including in silico, in vitro, and in vivo models [42,43].

The in silico method uses computational models to study the fate of phytonutrients in humans, similar to pharmaceutical drugs. One useful way to estimate the bioavailability of a novel phytonutrient or its metabolite is the computational method given by Lipinski’s ‘rule of five’. According to this, “for a compound to have good oral bioavailability, it should have a molecular mass of ≤500 Daltons, ≤5 hydrogen bond donors, ≤10 hydrogen bond acceptors, and a lipophilicity of ≤5 (expressed as logP)” [44].

In vitro studies often encompass the evaluation of three key parameters: 1. simulated gastrointestinal digestion, incorporating simulated digestion models; 2. absorption; and 3. biological activities, both assessed using cell lines. Simulated digestion models, depending on the study purpose and food type, may include oral, gastric, small intestine, and, in some cases, colonic fermentation stages. Various in vitro digestion models have been used in the literature that can be categorised as batch (or static) and continuous (or dynamic) models [45]. A batch model of digestion system is used more often for its convenience and cost-effectiveness. The simulated fluids are prepared to mimic human gastrointestinal digestion, and food products at each stage are incubated for a specified time at a specified temperature and pH. Owing to its simplicity, this method has been widely used in the literature with many modifications and variations, leading to inter-laboratory differences that make the results difficult to compare. To address this issue, a standard in vitro digestion model called INFOGEST has been established by a group of experts [46]. This model is widely adopted by researchers today, improving the comparability and reliability of results. However, despite their convenience, static digestion models may not accurately reproduce the dynamic, complex in vivo conditions that continuously change, along with the physical forces causing the breakdown of food particles. This limitation is addressed by dynamic digestion models such as the Dynamic Gastric Model (DGM) [47], the Human Gastric Simulator (HGS) [48], the commercial TNO™ gastrointestinal model TIM-1 [49], and the Simulator of the Human Intestinal Microbial Ecosystem (SHIME^®^) [50], which are computer-controlled to continuously monitor and regulate gastrointestinal conditions. These models incorporate continuous peristaltic contractions, enzymes, and digestive juice secretions and maintain appropriate pH, time, and temperature [49,50,51].

Different cell culture models are used to study intestinal absorption, transport efficiency, and bioactivity. The most commonly studied biological activities include antioxidant, vascular function, antiproliferative, antimicrobial, and anti-inflammatory effects. The MKN-28 (gastric cell) and Caco-2 (intestinal cell) cells have been frequently used, simulating the gastric and intestinal environments, respectively [52,53,54,55], while VSMC (vascular smooth muscle cells) and HUVECs (human umbilical vein endothelial cells) have been used to study vascular function [56,57,58]. Other cells such as HepG2 (human liver cancer cell), BRL-3A (rat liver cell), SH-SY5Y (human neuronal cell), and prostate cancer cells are being used to assess the antiproliferative and anticancer effects of anthocyanins [59,60].

Both animal and human studies involve tracking the concentration of phytonutrients and their metabolites in blood, urine, faeces, or other biological fluids over a specific period after consumption [61,62]. Biomarker assessments can examine the impact of phytonutrient consumption on specific biological markers or endpoints related to health outcomes [63]. However, with human studies, it is not always possible to study the digestibility and some biological activities due to ethical considerations. To address this gap, researchers commonly employ a combination of in vitro, in vivo, and in situ models [64,65,66,67]. These models allow for the exploration of absorption at different stages and the investigation of biological activities. For instance, the various stages of anthocyanin digestion, including oral, gastric, and intestinal processes, cannot be adequately examined through human or animal studies in isolation. Therefore, employing an in vitro digestion model as an additional step can provide insights into these stages. Similarly, inclusion of in vivo models can give information on absorption or bioaccessibility in different tissues and organs in addition to plasma, urine, and faeces. Therefore, a comprehensive phytonutrient bioavailability study includes digestion, absorption, metabolism, biotransformation, and elimination of anthocyanins in the system, and lastly, the ability to exert a beneficial physiological effect.

## 4. Metabolism of Anthocyanins in the Body

The absorption and metabolism of an anthocyanin mainly depend on its chemical structure, whether or not it is attached to a sugar moiety, the presence of methyl or acyl groups, the food matrix, the presence of other phytonutrients, the extent of food processing, and individual factors such as nutrition, health status, and genetics [21,26]. Previous studies have shown that absorption mainly takes place in the small intestine following enzymatic breakdown during gastrointestinal digestion [68]. However, recent findings have revealed a more complex picture where anthocyanins undergo biotransformation into different metabolites, including glycosides, esters, and polymers [69,70,71,72]. This transformation does not exclusively occur in the small intestine but also involves enzymatic and gut microbial modifications, thereby continuing their journey into the large intestine [73,74]. In addition to this, several in vivo studies have shown the detection of anthocyanins and their metabolites in different tissues and organs, including the brain, liver, kidneys, and pancreas [75]. Hence, the exploration into the metabolism of anthocyanins is still ongoing and not fully established.

The section below will briefly discuss the digestion, absorption, and metabolism of anthocyanins at each stage in the gastrointestinal tract.

### 4.1. Oral Cavity

Anthocyanins undergo a process of bioactivation and partial degradation, primarily influenced by β-glucosidase activity. This process is significantly affected by the oral microbiota as well as oral epithelial cells and other salivary enzymes. However, substantial inter-individual differences exist in β-glucosidase activities [76]. In an ex vivo study where the oral metabolism of anthocyanins from five different berries was assessed using saliva from 14 healthy subjects, partial degradation of anthocyanins of up to 50% was observed. It showed that the glycosides of delphinidin and petunidin were more susceptible to degradation compared to malvidin, peonidin, pelargonidin, and cyanidin [77]. Additionally, di- and trisaccharide forms were found to be more resistant to degradation than monosaccharides [77]. However, the brief exposure time in the oral cavity makes it difficult to accurately assess the metabolism affected by enzymes and the oral microbiome.

### 4.2. Gastric Digestion

The gastric environment, characterised by a low pH ranging from 1.5 to 2, is well suited for anthocyanins as they are highly stable under acidic conditions [78]. Some studies have shown a surprisingly high recovery rate, even higher than 100% [79,80]. This exceptional stability arises from the acidic conditions within the stomach, which facilitate the conversion of anthocyanins into red flavylium cations. These flavylium cations absorb a greater amount of light at 520 nm compared to native anthocyanins, leading to an overestimation of anthocyanin species [80]. Upon ingestion, the anthocyanins are readily detected in the plasma as well as urine in their intact form, along with methylated, glucuronidated, and sulphoconjugated forms, during the gastric phase [68,81]. This rapid appearance of anthocyanins in the plasma is attributed to their capacity to pass through the gastric mucosal barrier through bilirubin translocation enzymes or bilitranslocase-mediated mechanisms, with no detection of anthocyanin metabolites or aglycones in the stomach [67,82]. Bilitranslocase has a higher affinity for the parent anthocyanins glycosides than aglycones and is considered an important delivery tool for the systemic circulation to exert acute effects. Further, an in-situ study on rats administered with anthocyanins into the stomach showed approximately 25% of absorption for anthocyanin monoglycoside from the stomach [67].

### 4.3. Small Intestinal Absorption

The pH of the small intestine, ranging from 6 to 8, significantly reduces the stability of anthocyanins and transforms the red coloured flavylium cation into colourless or nearly colourless compounds such as chalcones, quinone bases, and carbinol pseudobases [83,84]. While the primary site of absorption is in the jejunum, a small portion is absorbed in the duodenum, and negligible absorption occurs in the ileum and colon [67,68].

The glycosides of anthocyanins undergo rapid and efficient absorption within the small intestine, which is then followed by rapid metabolism and excretion in the bile and urine as both the intact form and metabolised derivatives (methylated, glucuronidated, or sulphated) [67,68,85]. Two potential mechanisms have been proposed for intestinal absorption: one through active transportation facilitated by multiple transporters expressed in the intestinal epithelial cells, such as specific glucose transporters (SGLT1 and GLUT2), while the other involves intracellular hydrolysis of anthocyanins by brush border enzymes, such as lactase-phlorizin hydrolase, followed by passive diffusion of the aglycone [86,87].

An in-situ study involving the perfusion of pure anthocyanin glycosides and anthocyanin extracts from blackberry and bilberry into rats showed rapid and efficient absorption up to 22.4%, depending on the chemical structure [68].

In vitro investigations commonly utilise the Caco-2 cell line derived from a human colon adenocarcinoma, which serves as a well-established model for simulating the absorption process in the small intestine. Even though these cells are of colonic origin, they exhibit functional and morphological characteristics typical of small intestinal cells. This model is used to investigate the absorption rate and transport efficiency of anthocyanins [88]. For example, the transport efficiency of blueberry anthocyanins averaged 3–4%, while the absorption rates of anthocyanin extracts (glucosides of Mv, Pt, Peo, Del, and Cy) from grape/blueberry extract were found to be as low as 0.005–0.06% [89].

### 4.4. Gut–Microbiota Interaction and Colonic Metabolism

Recent research has revealed that phytonutrients have prebiotic potential, enabling them to interact with the gut microbiota to produce beneficial metabolites as well as modulate the microbiome composition [90,91]. Unabsorbed anthocyanins, along with their metabolites, interact with the intestinal microbiota and serve as substrates for several enzymes present in the small intestine, liver, colon, and kidney [92]. The majority of intestinal bacteria, including *Lactobacillus* spp. and *Bifidobacterium* spp., possess β-glucosidase activity, enabling the conversion of anthocyanins to more bioavailable forms. This can also occur by the process of conjugation with the addition of methyl, hydroxyl, sulphuric, or glycosidic groups [92]. Therefore, it is crucial to consider these breakdown products when assessing the bioavailability of anthocyanins. Some highly bioactive derivatives include aldehydes and phenolic acids, mainly vanillic acid, protocatechuic acid, phloroglucinol aldehyde, and gallic acid, which are absorbed by epithelial tissues, excreted to the jejunum via bile, and recycled through the enterohepatic circulation system [92,93]. Other phenolic acid derivatives reported as produced by in vitro digestion comprising a chemostat (continuous fermentation system) include cinnamic acid, chlorogenic acid, caffeic acid, quercetin arabinoside, kaempferol 3-rhamnoside, syringetin-3-galactoside, hippuric acid, and rhamnetin [94]. In vitro fermentation using microbiota from pig cecum showed that all anthocyanins were hydrolysed within 20 min, which was evident by decreased parent anthocyanins and increased anthocyanin degradation products [95]. These phenolic acid metabolites have been shown to have good anti-inflammatory, antioxidant, and antitumour properties [96].

Furthermore, anthocyanins are able to profoundly modulate the gut microbial composition. In vitro, animal and human studies have demonstrated that anthocyanins stimulate the growth of beneficial bacteria, such as *Lactobacillus* spp. and *Bifidobacterium* spp., while inhibiting the growth of harmful bacteria such as *Salmonella typhimurium* and *Staphylococcus aureus* [97]. In addition to this, anthocyanins have also been shown to enhance the proliferation of *Lactobacillus acidophilus*, *Bifidobacterium adolescentis*, *Bifidobacterium infantis*, and *Bifidobacterium bifidum* [92,98]. These beneficial bacteria have the ability to produce beneficial metabolites such as short-chain fatty acids, compete for substrates, and exert antimicrobial effects [92]. In another study, anthocyanin-rich black raspberry extract was fermented with the gut microbiota of infants, human adults, mice, and rats to investigate its effects on gut microbial diversity and microbial community structure [71]. An increase in alpha diversity was observed solely in the adult and rat microbiota, while it remained relatively stable in the infant and mouse microbiota, indicating that anthocyanins have varied effects on different microbiota sources. Higher alpha diversity, as evidenced in the literature, has been linked to better overall health outcomes compared to lower diversity, which is associated with chronic diseases such as inflammatory disorders [99,100]. Similarly, significant variation in beta diversity was observed across all samples, along with notable differences in microbial community structures in all samples except for the mouse microbiota, underscoring the inter-individual differences in microbial community composition across these sources [71]. Furthermore, the black raspberry extract inhibited the growth of the pathogenic genus Escherichia/Shigella and promoted beneficial bacteria, particularly Fusicatenibacter and Lachnoclostridium, in all microbiota sources [71]. These results validate the ability of anthocyanins to modulate gut microbiota composition and diversity, thereby contributing to potential health-promoting effects, including reducing insulin resistance, obesity, inflammation, and cardiovascular issues [101,102,103].

## 5. Effects of Food Matrix on Anthocyanin Bioavailability

The concept of a food matrix encompasses not only the physical and chemical constituents of a given food but also the intricate molecular relationships and complex interactions between these components [104]. These interactions play a crucial role in shaping how a food is digested, metabolised, and exerts a beneficial effect upon consumption. With the increase in phytonutrient-based nutraceuticals on the market, a pressing question emerges: Are these concentrated forms of phytonutrients truly superior to whole foods? Addressing this query necessitates a comprehensive understanding of the food matrix’s impact on bioavailability to discern the advantages/disadvantages of consuming isolated forms of phytonutrients.

### 5.1. Effects of Food Matrix on Bioaccessibility, Digestion, and Absorption

The digestion and absorption capacities of anthocyanins from different food sources are summarised in Table 1. An in vitro study conducted to determine the effect of food matrix on anthocyanin digestibility, comparing whole red cabbage and anthocyanin rich-extract from red cabbage, found that anthocyanin stability during the digestion process was strongly dependent on the food matrix [105]. The recovery of anthocyanins after digestion from red cabbage was reported to range from 18 to 113%, whereas for anthocyanin-rich extract, it was from 3 to 31%. Consequently, the whole red cabbage digesta (both gastric and intestinal) showed significantly higher antioxidant properties compared to digests from anthocyanin-rich extracts.

Another study examined the impact of the food matrix, particularly glucose, proteins, and starch, on purple-fleshed sweet potatoes and red wine [53]. The study revealed that the anthocyanin levels in purple-fleshed sweet potatoes decreased by 27–43% without matrix components and by 22–31% with matrix components during digestion. A similar trend was observed in red wine, where the reduction in anthocyanins was 49–52% without matrix components and 30–45% with added matrix components. In addition to this, it was revealed that the presence of glucose and proteins reduced the transport efficiency, while the presence of starch did not have any effect [53]. These findings suggest that different food components may exert varying effects on anthocyanin absorption.

Findings from two separate studies investigating the bioavailability of anthocyanins in blueberries, one in purified form and the other using freeze-dried fruit, revealed opposite results [94,106]. In the study focusing on purified anthocyanins from wild blueberries, in vitro intestinal digestion caused a degradation of 42% of anthocyanins, that is, recovering 58% of total anthocyanins [106]. Conversely, in another study, when frozen blueberries were subjected to in vitro intestinal digestion, the total anthocyanin content recovered was only 15% [94]. However, direct comparisons are needed to confirm whether purified extracts are superior to whole fruit in terms of digestion in order to eliminate differences in doses, inter-laboratory variations, and blueberry cultivars.

The general notion is that the food matrix may exert a protective effect against the degradation of anthocyanins until they reach the intestinal digestion environment, thereby enhancing anthocyanin stability against various enzymes and digestive fluids. This is because the anthocyanin entrapped in the complex food matrix is released slowly into the system compared to its isolated counterparts. This protective effect can also be achieved by encapsulation techniques. For example, a study found that purified blueberry anthocyanins released after 2.5 h of in vitro digestion were lowest when encapsulated in microcapsules made of soy protein isolate (27%) followed by gelatine (28.7%), arabic gum (54.2%), and maltodextrin (63.0%), compared to anthocyanins that had not been encapsulated (70.9%) [107]. In addition to this, food composition, processing techniques involved, and anthocyanin structure can also affect bioavailability. Therefore, each phytonutrient behaves or interacts differently with the other food components, influencing their stability during digestion and subsequent absorption or transportation across the intestine.

### 5.2. Effects of the Food Matrix on the Bioactivity of Anthocyanins

#### 5.2.1. Antioxidant Properties

It has been well-documented in the literature that anthocyanins inherently possess antioxidant properties, as evident from several in vitro studies where they consistently exhibit antioxidant efficacy irrespective of their form—whether purified, in crude extracts, or within whole foods [54,58,59,60,78,94]. These studies, summarised in Table 1, predominantly employ cell models to assess antioxidant activity. Examples include investigations into the antioxidant effects of delphinidin chloride on HUVEC cells [58], cyanidin-3-glucoside and protocatechuic acid on SH-SY5Y cells [59], anthocyanin extract from purple rice on BRL-3A cells [60], commercial Chinese red wine on Caco-2 cells [54], and frozen wild blueberries on CRL 1790 and HT 29 cells [94]. This remarkable antioxidant capacity is attributed to the structural characteristics of anthocyanins, particularly their conjugated rings and phenolic hydroxyl groups, as substantiated by existing research [108].

Evidence from animal studies (Table 2) includes the intraperitoneal administration of 100% cranberry juice in hamsters, which demonstrated notably elevated antioxidant properties across various organs, including the liver, heart, brain, bladder, and kidney, compared to baseline [109]. The anthocyanin content in cranberry juice was noted to be only 31% of its total polyphenol content, suggesting that the observed effect could be attributed to the presence of other polyphenols and components along with anthocyanins [109]. Another study investigated the impact of the food matrix in black currant on antioxidant activity in Watanabe heritable hyperlipidaemic rabbits [110]. The treatments included black currant juice concentrate, pure anthocyanins derived from black currants (63% and 79% purity, respectively), and a control group. While the Trolox equivalent antioxidant capacity assay showed no significant differences in antioxidant activity among the samples, the ferric reducing ability of plasma assay showed a significant increase in antioxidant activity in the group fed with black currant juice compared to rabbits fed with purified anthocyanins between 0.25 and 2 h after dosage [110]. The author attributes this effect to other components present in the juice besides anthocyanins, or it could also be caused by interactions between anthocyanins and the food matrix [110].

Human clinical trials (Table 3) investigating the antioxidant effects of grape juice and wine consumption, revealed a higher antioxidant activity in grape juice compared to wine, potentially attributed to its higher glucose content [111]. The presence of sugar is presumed to have a synergistic effect in the juice and may have a protective action on anthocyanin by increasing its stability, thereby increasing its antioxidant properties, as shown by other studies [112,113]. Further, the consumption of acai pulp demonstrated significantly increased antioxidant effects in plasma compared to acai clarified juice [114], reinforcing the hypothesis that whole solid foods may offer greater health benefits than products lacking essential constituents.

Notably, the antioxidant nature of anthocyanins is highest in either gastric or intestinal digests compared to undigested forms, with the gastric environment being more favourable [78,115]. This indicates that the digestion process has a prominent role in enhancing the antioxidant property. Therefore, studying the bioactivity of these compounds directly on the cells without subjecting them to the digestion process may not accurately reflect the outcomes observed within the human body.

#### 5.2.2. Anti-Inflammatory Properties

Anthocyanins are widely studied for their ability to alleviate inflammation in animals, as depicted in Table 2, and are considered a potential therapeutic option for inflammatory bowel diseases. Anthocyanin-rich extracts from black rice [116], bilberry [103], blueberry [117], and Portuguese blueberry [118] all showed anti-inflammatory effects in mice with induced colitis. This is achieved by their ability to inhibit the pro-inflammatory cytokine secretions of TNF-α, IL-6, and IL-8, improving intestinal permeability, colonic MPO activity, and mRNA expression [119]. Remarkably, Portuguese blueberry exhibited not only significant anti-inflammatory effects but also a remarkably high antioxidant effect in mice, surpassing the effectiveness of the commonly used aminosalicylate drug, even at a concentration 30 times lower [118]. Furthermore, purified anthocyanins from mulberry [120], crude anthocyanin rich extracts of blueberry [117], purple yam [121], and black rice [116] supplemented diets in mice reduced colonic inflammation, oxidative stress, and tissue damage and improved intestinal barrier functions. Purified anthocyanin, cyanidin-3-O-β-glucoside, improved liver function by inhibiting liver fibrosis and activating hepatic stellate cells [122].

Concerning whole foods, two studies on freeze-dried purple potatoes [123,124] and one study on lingonberry juice [125] have shown that these food sources reduce inflammation in a mouse model of colitis. However, interestingly, in one study where male Fischer 344 rats were fed with anthocyanin-rich grape–bilberry juice, no change in immune function or improvement of inflammation markers was observed [126]. Although all forms of anthocyanins, including purified, crude extracts, and anthocyanin-rich whole foods, have demonstrated anti-inflammatory effects in animal models, there are currently no direct comparison studies confirming which form of anthocyanins may be most beneficial in terms of anti-inflammatory effects.

Lastly, a randomised human clinical trial, as summarised in Table 3, examined the effect of pigmented potatoes, namely, white, yellow (rich in carotenoids), and purple (rich in anthocyanins), on oxidative stress and inflammation markers [127]. While consumption of both yellow and purple potatoes reduced oxidative stress and plasma IL-6 in men, purple potato consumption also lowered the C-reactive protein concentration in plasma significantly compared to white potatoes. This effect was attributed to the presence of anthocyanins, which is almost undetectable in white and yellow potatoes.

#### 5.2.3. Anticancer Properties

While phytonutrients in general are widely recognised for their anticancer properties, the evidence supporting these claims is primarily derived from cell models. Unfortunately, there is a lack of comprehensive data from animal and human clinical studies, often due to challenges related to dosing and bioavailability limitations.

In in vitro studies (Table 1), purified anthocyanins from red wine showed anticancer properties in gastric (MKN-28) [52] and intestinal (Caco-2) [57] cell lines, while crude anthocyanin extract from black raspberry failed to show anticancer effects on prostate cells [128]. This suggests that the anticancer properties of anthocyanins may depend on their source as well as the target region.

Further, two studies have consistently shown that freeze-dried blueberry and black raspberry dietary supplementation in rats effectively reduced mammary tumour volume up to 69% [129,130]. In a separate investigation, rats were fed commercial anthocyanin-rich extracts from bilberry, chokeberry, and grapes to assess their anticancer effects. Interestingly, only chokeberry and bilberry exhibited significant anticancer effects against colonic cancer, while grape extract did not produce the same outcome [6].

#### 5.2.4. Obesity and Antidiabetic Properties

Two animal studies (Table 2) conducted on mice using bilberry air-dried powder [103] and freeze-dried jaboticaba peel powder [131] demonstrated improved insulin sensitivity and regulated glucose and cholesterol levels. Furthermore, an investigation into the impact of jaboticaba peel powder on obesity in mice revealed reduced insulin resistance, increased HDL cholesterol levels, and no discernible effects on energy intake, weight gain, or body fat [131]. Similarly, in mice, incorporating bilberry into a high-fat diet partially prevented the elevation of serum cholesterol, glucose, and insulin levels, concurrently reducing inflammation, while weight gain remained unchanged [103]. These findings from animal studies align with those of a human clinical trial (Table 3), in which the antidiabetic effect of commercially available anthocyanin capsules, purified from bilberry and blackcurrant, was investigated in 58 diabetic adults [132]. The consumption of anthocyanin capsules showed improvements in dyslipidaemia, prevented insulin resistance, and increased antioxidant effects in plasma [132]. From the above two studies, it can be noted that bilberry could serve as an effective antidiabetic agent when consumed in both purified (as capsules) and whole fruit forms. However, further investigation is required, considering equivalent doses and the use of the same clinical model.

#### 5.2.5. Antiplatelet Effects

In a comparative study, the combined use of grape seed extract and grape skin extract exhibited a heightened antiplatelet effect on human platelets, both in vitro and in ex vivo feeding studies on dog platelets, in comparison to when these extracts were employed individually [133]. This enhanced effect is attributed to the interaction of specific polyphenolic compounds found in grape seed with those in grape skin, suggesting an additive influence that contributes to the observed antiplatelet effect [133].

#### 5.2.6. Other Effects

Other widely studied biological effects of anthocyanin extracts and anthocyanin-rich foods include enhanced vascular function [8,134,135,136,137], cardioprotective effects [138], antimicrobial activity [11,139,140], osteoprotective [141], neuroprotective [142], and gut health-promoting properties [98].

**Table 1:** Literature on the bioavailability of anthocyanins—in vitro studies.

ACN Form	Anthocyanin Source	Digestion	Absorption (Transport Efficiency)			Bioactivity/Health Effects	Ref.
		Stability (S)/recovery (R)/degradation (D)	Dose	Cell model/dose	Transport efficiency		
Pure	Commercial delphinidin chloride		1 nM to 100 μM.	HUVEC	ND	Antioxidant	[58]
Pure	Cy-3G, and PA	ND	NA	SH-SY5Y	ND	Antioxidant and neuroprotective	[59]
Purified	Chinese Vitis davidii red wine	ND	200 μM/3 h	Caco-2; MKN-28	3–5%; 4–9%	Anticancer	[52]
Purified vs. whole	Purple-fleshed sweet potato	D: 27–43% (no food matrix) 22–31% (food matrix)	150 µL	MKN-28 (3 h) Caco-2 (2 h)	5% 8%	ND	[53]
Purified vs. whole	Red grape	D: 49–52% (no food matrix) 30–45% (food matrix)	ND			ND	[53]
Purified	Strawberry	ND	Pg3G: 10 μg/mL Pg3R: 50 μg/mL	Caco-2	Pg3R: 1.13%; Pg3G 0.28%	ND	[143]
Purified	Wild Chinese blueberries	D: 42%	50 mg/mL; 2 h	Caco-2	1.59% to 4.22%	ND	[106]
Purified	*Hibiscus sabdariffa* L.	Gastric: 49% d3s, 70% c3s Intestine: 3% d3s, 10% c3s	ND			Antimicrobial	[140]
Crude extract	Red grape/bilberry	MO-fermented ACNs	50 μmol/L	Co-culture: Caco-2 and HUVECs	ND	Anti-inflammatory and antiadhesive	[56]
Crude extract	Raspberry	~5% in serum; ~70% in GIT		ND		ND	[144]
Crude extract	Blueberry		50 μg/mL	Caco-2	∼3–4%	ND	[55]
Crude extract	Cornelian cherry	Stomach: 107.23% Intestine: 26.46%				Antioxidant	[78]
Crude extract	Purple rice	76% degraded		BRL-3A		Antioxidant	[60]
Crude extract (separate)	Blackberries, red apples, strawberries, and grapes.	S: gastric: 114–179% Intestine: 1.6–82.5%		ND		ND	[79]
Crude extract	Black raspberries	ND	1 mg/mL	6 prostate cancer cells: LNCaP, LAPC-4, VCaP, 22Rv1, PC-3, and C4-2		No effect on prostate cancer	[128]
Concentrate	Commercial Montmorency tart cherry	ND	PCA: 32 μM; VA: 4 μM	VSMC		Vascular protective	[57]
Extract	Commercial Bilberry and blackcurrant	Gastric > oral > intestine	0.18, 0.37, 0.75, and 1.5 μg/mL	Caco- 2	ND	Anti-inflammatory	[145]
Extract	Commercial Black currant	ND	180.3 ± 19.3 µmol/L; 20 min	Caco-2	11% at 20 min; Del > Cy	ND	[146]
Juice	Pomegranate	R: 2.4–15.3%		ND		ND	[147]
Wine	Commercial Chinese red wine	D: 14.5–28.3%	0.5 ml	Caco-2	2.08–24.01%.	Antioxidant	[54]
Wine	Commercial red wine	R: serum: 3.7%; colon: 37%		ND		ND	[115]
Freeze-dried powder	Purple carrots (PC) and purple potatoes (PP)	R: PC: 45; PP: 71.8%	200 µg/mL (semi-purified extract)	Caco-2 BBe; THP-1	PC: 6%; PP: 36%	Anti-inflammatory	[119]
Frozen	Wild blueberries	R: gastric: 97%; intestine: 17%; fermented: 1.5%	10, 25, 50, 75, or 100 μg/ml	CRL 1790; HT 29	ND	Antioxidant and anticancer	[94]

W: whole food; E: extract; MKN-28: human gastric cancer cell; Caco-2 and HT-29: human colorectal cancer cell; HUVEC: human umbilical vein endothelial cell; SH-SY5Y: human neuronal cell; BRL-3A: rat liver cell; THP-1: human monocytic leukaemia cell; CRL-1790: human foetal colon epithelial cell; HepG2: human liver cancer cell; VSMC: vascular smooth muscle cells; Cy-3G: cyanidin-3-glucoside; Pg3R: Pg3G: pelargonidin-3-glucoside; pelargonidin-3-o-rutinoside; Mv-3-glu: malvidin-3-glucoside; Pt-3-glu: Dp-3-glu: delphinidin 3-O-glucoside; petunidin 3-O-glucoside; PCA: protocatechuic acid; VA: vanillic acid; PC: polyphenolic content; ACN: anthocyanin; GIT: gastrointestinal tract; TAC: total anthocyanin content; TNF-α: tumour necrosis factor alpha; Nrf2: nuclear factor erythroid 2–related factor 2; NF-κB: nuclear factor kappa-light-chain-enhancer of activated B cells; IL-8: interleukin-8. Food or food products that have undergone single-stage extraction, such as solvent extraction, are classified as crude extracts, whereas those that have undergone more than three stages of extraction, including chromatographic technique, solid-phase extraction, and adsorption resins, are classified as purified forms of anthocyanins.

**Table 2:** Literature on the bioavailability of anthocyanins—animal studies.

ACN Form	ACN Source	Treatment and Dose	Model	Bioavailability	Bioactivity	Ref.
>99% pure Cy-3G	Commercial (blackberry)	500 mg/kg gavage (n = 21); C3G at 1 mg/kg via tail vein injection (n = 40)	C57BL6J mice	Systemic bioavailability: parent Cy-3G and total ACNs were 1.7% and 3.3%, respectively.	NA	[7]
>96.5% pure Cy-3G	Black rice	3 groups Control (vehicle) olive oil only, 10% CCl4 in olive oil, CCl4 plus 800 mg/kg of C3G.	Male C57BL/6 mice (8 wks old)	Serum and liver: no Cy-3G but PCA detected, which was confirmed to be a metabolite of C3G.	Liver function: Cy-3G with CCL4 inhibited liver fibrosis and the activation of hepatic stellate cells.	[122]
Purified Cy-3G	Commercial	2 groups (n = 22) Control (0.2 mL of PBS), PBS with 668 nmol Cy-3G at specific time points (0.25, 5, 10, 15, 20 min)	Male Wistar rats (15 wks old); BW: 293–390 g	Plasma: Cy-3G > Mv-3G > Peo-3G > Pel-3G (AUC) Brain: Cy-3G > Pet-3G > Peo-3G (AUC) Liver: Cy-3G, Peo-3G, Pet-3G Kidney: Cy-3G, Peo-3G, Pet-3G Urine: Cy-3G, Peo-3G	NA	[148]
Purified Cy-3G	Commercial	Cy-3G (50 mg/kg BW), PCA (5 mg/kg BW), or Cy-3G (50 mg/kg BW) plus PCA (5 mg/kg BW). 14 days; 4 weeks.	ApoE−/− mice	NA	PCA: antiatherogenic effect by inducing ABCA1 and ABCG1 expression in macrophages.	[149]
Purified ACNs	Blackberry and bilberry	In situ perfusion, 45 min Purified ACNs: 9.2 nmol/min, blackberry ACNs: 9.0 nmol/min, and bilberry ACNs: 45.2 nmol/min.	Male Wistar rats, ~200 g BW	Small intestine: rate of absorption: 10.7 to 22.4% Plasma and urine: native cyanidin 3-glucoside was recovered in urine, and plasma from the aorta and mesenteric vein along with methylated and/or glucuronidated derivatives. Bile: cyanidin 3-glucoside and its methylated derivatives.	NA	[68]
Purified	Commercial delphinidin chloride	sRANKL-induced osteoporosis model mice: (n = 17), 10 mg/kg/day. 17 days Ovariectomised (OVX) mice (n = 24) Control, 1 mg/kg, 3 mg/kg, 10 mg/kg; 28 days (n = 6 each)	Female C57BL/6 mice (7 wks old)	NA	Osteoprotective: ↓ bone loss in both RANKL-induced osteoporosis and OVX mice by suppressing the activity of NF-kB, c-Fos, and NFATc1, master transcriptional factors for osteoclastogenesis.	[141]
Purified	Blueberry and bilberry	Study 1: 5% blueberry powder and an AIN-93 M diet for 10 days. Study 2: 10 mg bilberry ACNs in 10% dimethyl sulphoxide 2 h.	Female athymic nude mice (5–6 wks old)	Plasma: recovered 55–95% of ACNs, 63–100% of anthocyanidins. Lungs: Cy is readily detected.	NA	[75]
Purified	Commercial (blackcurrant) (BC)	4 groups (n = 5 each) Control, 63% BC juice concentrate, and 79% pure ACN.	Watanabe heritable hyperlipidaemic rabbits (6 wks old)	Plasma: detected at tmax 30 min Urine: 0.035% in the first 4 h	Antioxidant activity: TEAC: no effect; FRAP: BC juice > pure ACNs	[110]
Purified	Mulberry ACN (MAS)	4 groups, oral, 17 days Control, DSS-fed, DSS + 100 mg/kg BW of MAS, DSS + 200 mg/kg BW of MAS.	Male C57BL/6J mice (6–7 weeks old; 20 ± 2 g BW)	NA	Weight loss: *p* < 0.001; ↓ Disease activity index Anti-inflammatory and ↓ gut dysbiosis	[120]
Crude extract	Black rice anthocyanin extract (BRAE)	3 groups (n = 10 each) Control, DSS, and DSS + BRAE (200 mg/kg/day) by gavage.	The DSS murine model of colitis Male C57BL/6 mice (8 wks old)	NA	Inflammation: BRAE ↓ DSS-induced colonic inflammatory phenotypes, maintained colon length in mice, ↓ intestinal permeability, and improved intestinal barrier dysfunction in mice with colitis. Gut: BRAE ↓ inflammatory bacteria, and ↑ anti-inflammatory probiotics, including *Akkermansia* spp.	[116]
ACN-rich extracts (AREs)	Commercial bilberry, chokeberry, and grape	2 groups, 14 weeks Control diet and control diet with AREs.	Fischer 344 male rats (4 wks old)	Serum: detectable below quantifiable levels Urine: 7.8 mg/L to 23.6 mg/L Faeces: up to 2.0 mg/L in bilberry and chokeberry, and 0.7 mg/L in grape.	Colon cancer: bilberry ARE (*p* = 0.008) and chokeberry ARE (*p* = 0.015); grape ARE has no effect	[6]
Crude extract	Bilberry	Study 1: 100 mg/kg BW and vehicle control group after 12 h of starvation. (n = 5 each) Study 2: 500 mg/kg body weight (n = 5) without prior starvation. Study 3: 0.5% by weight. (n = 10) and control.	Male C57BL/6 mice	Plasma: total ACNs peaked at 1.18 ± 0.3 μM after 15 min; Urine: 1.88%. Tissues: detected in the liver, kidney, testes, and lung, with a maximum of 605, 207, 149, and 116 pmol/g, respectively. not detectable in the spleen, thymus, heart, muscle, brain, white fat, or eyes.	NA	[150]
Crude extract (32%)	Blueberry	5 groups (n = 10 each), 6 days 1 vehicle group, a TNBS control group (inducing colitis), and three ACN groups receiving daily doses of 10, 20, and 40 mg/kg of ACNs.	Female C57BL/6 mice	NA	Anti-inflammatory (colon) also prevented weight loss, improved diarrhoea scores, morphology, and histology,	[117]
Crude extract	Mulberry	6 groups (n = 10 each) oral gavage; 8 weeks. 1. Young rats—normal diet and 300 mg/kg mulberry extract 2. Aging rats—normal diet, 100, 200, and 300 mg/kg mulberry extract.	Male Sprague-Dawley rats at 8 and 80 wks of age.		Cardiovascular protection alleviated endothelial senescence, oxidative stress in the aorta, and improved eNOS function in aging rats.	[151]
Crude extract	Purple yam	7 groups (n = 10 each) Control; TNBS; TNBS with 75 mg/kg 5-aminosalicylic acid; 20, 40, and 80 mg/kg ACNs; 75 mg/kg 5-aminosalicylic acid without TNBS induction; and 80 mg/kg ACNs without TNBS induction.	Male C57BL/6 mice (6 wks old); colitis induced by TNBS intra-rectally.	NA	Anti-inflammatory: the TNBS-A80 group showed a stronger protective effect. TNF α, interferon γ in serum ↓. All doses of ACN reduced iNOS concentrations. Body weight: TNBS-A80 rapid weight recovery from day 3	[121]
ACN-rich extract	Portuguese blueberries	4 groups (n = 10 each) Noncolitic control, TNBS-colitic control, TNBS-induced rats treated 10 mg/kg with ARF, and TNBS-induced rats treated with 100 mg/kg 5-5-aminosalicylic acid.	Male Wistar rats (4 weeks old); 2,4,6-trinitrobenzenesulphonic acid (TNBS)-induced colitis rat model.	NA	Anti-inflammatory, ↓ leukocyte infiltration, antioxidant activity ARF > 5-ASA.	[118]
Anthocyanin-extract	Commercial (bilberry)	3 groups (n = 50) Control diet and diet with 1% and 10% bilberry extract.	Female BALB/c mice (20–22 g BW) colonic cancer induced by azoxymethan (AOM) and DSS (3 or 5%)	NA	Anticancer and anti-inflammatory	[152]
ACN extract powder	Commercial (blackberry)	2 groups (n = 6 each) Control diet, 15 g BB per kg diet (14.8 mmol ACNs per kg diet), 15 days	Male Wistar rats, 250 g BW	Stomach: 91.7%, jejunum: 80.2%, kidney: 66.1%, liver: 13.2%, brain: 84%, plasma: 41.7% urine: 0.19 ± 0.02%.	NA	[153]
100% juice	Commercial (cranberry) CJ	2 groups (n = 7 each) administered i.p. daily for 7 days. Control, 1 mL of CJ	14 Syrian golden hamsters	Liver, kidney, heart, bladder, and brain	Antioxidant activity	[109]
Juice	Lingonberry (LBJ)	5 groups, 3 weeks Control, control + 33% LBJ, IR, IR + 33% LBJ, and IR + 20% LBJ	Sprague–Dawley rats Ischemia–reperfusion-induced (IR)	NA	Anti-inflammatory: kidney	[125]
Juice	Grape–bilberry (80:20)	2 groups (n = 24 each), 10 weeks Control and ACN-rich juice.	Male Fischer 344 rats (10 wks old)	Plasma and urine: low nanomolar concentrations. Small intestine: 570 ng/g	No effect on inflammation (serum)	[126]
Individually quick freeze-dried	Tart cherry	2 groups (n = 9 each) 1% tart cherry diet, 10% tart cherry diet (n = 9)	Male Wistar rats (6 wks old)	Tissues: the highest total ACNs found in the bladder and kidney for both groups, followed by the liver, heart, and brain.	NA	[154]
Air-dried powder	Bilberry	3 groups (n = 20) Low-fat diet (10%), high-fat diet (46%), and bilberry powder-supplemented high-fat diet (20% *w*/*w*).	Male C57BL/6N mice (8 wks old)	NA	Weight: no effect Hepatoprotective Metabolism: partially prevented the increase in serum cholesterol, glucose, and insulin levels.	[103]
Baked and freeze-dried	Purple-fleshed potato (PFP)	3 groups Control (AIN-93G diet) and 15% and 25% PFP diet.	The DSS murine model of colitis	NA	Anti-inflammatory (colon) ↓ gut dysbiosis	[123]
Baked and freeze-dried	Purple-fleshed potatoes	3 groups Control diet, 20% PFP supplemented diet, microbiota-ablated group.	Four-week-old male mice (C57BL6)	NA	Anti-inflammatory	[124]
Freeze-dried powder	Blackberry	2 groups (n = 18 each) Control diet and control diet supplemented with 200 g/kg blackberry powder plus 20 g/kg citric acid, 8 days	Male Wistar rats BW: ~170 g	Plasma: NIL Urine: cyanidin: ~0.26%; malvidin: 0.67% Caecal: recovered low amounts of glucosides and cyanidin	NA	[155]
Freeze-dried powder	Jaboticaba peel	5 different diets: (n = 8 each) Standard AIN-93G diet with 12% protein, modified AIN-93G high-fat diet (HF diet) with 12% protein and 35% lipids, and 3 groups of high-fat diet supplemented with 1%, 2%, and 4% freeze-dried jaboticaba peel powder.	Swiss male mice and Sprague-Dawley males	NA	Obesity: reduced insulin resistance. 2% FJP ↑ HDL-cholesterol levels by 41.65%. compared to the HF control freeze-dried No effect on energy intake, weight gain, and body fat.	[131]
Freeze-dried powder	Blueberry (BB) and black raspberry (BRB)	3 treatments, 6 groups Control diet, 5% *w*/*w* BB powder, 5% *w*/*w* BRB powder.	Female ACI rats (6 wks old)	NA	Anticancer (mammary) BB diet: 50.7% reduction; BRB diet: 42.4% reduction.	[129]
Freeze-dried powder	Blueberry and black raspberry	4 groups 1. AIN-93M diet (n = 25), BB diet (2.5% wt/wt) (n = 19), BRB diet (2.5% wt/wt); (n = 19), ellagic acid diet (400 ppm) (n = 22)	Female ACI rats (7–8 wks old)	NA	Anticancer (mammary) Tumour volume reduction BRB: 69%, BB: 40%.	[130]
Freeze-dried powder	Blueberry	Control, 2% (*w*/*w*) BB. 8 wks,	Neutered male Yorkshire X Landrace pig (32–41 days old)	Brain: detected 279–432 fmol/g of tissue in the brain.	NA	[156]

AIN: American Institute of Nutrition; Cy-3G: cyanidin-3-glucoside; Pg3R: Pg3G: pelargonidin-3-glucoside; pelargonidin-3-o-rutinoside; Mv-3-glu: malvidin-3-glucoside; Pt-3-glu: Dp-3-glu: delphinidin 3-O-glucoside; petunidin 3-O-glucoside; PCA: protocatechuic acid; VA: vanillic acid; PC: polyphenolic content; ACN: anthocyanin; BW: body weight; HFD: high-fat diet; LFD: low-fat diet; DSS: dextran sulphate sodium; NO, MPO: myeloperoxidase, IL-12: interleukins-12, and IFN-γ: interferon-gamma; SAA: serum amyloid A; MCP1: monocyte chemoattractant protein-1; JNK: c-Jun N-terminal kinase; COX: cyclooxygenase; NO: nitric oxide; TNF-α: tumour necrosis factor alpha; IL-8: TNBS: 2,4,6-trinitrobenzene sulphonic acid; eNOS: endothelial nitric oxide synthase; TEAC: Trolox equivalent antioxidant capacity; FRAP: ferric reducing ability of plasma; ↓: decreased; ↑: increased. Food or food products that have undergone single-stage extraction, such as solvent extraction, are classified as crude extracts, whereas those that have undergone more than three stages of extraction, including chromatographic technique, solid-phase extraction, and adsorption resins, are classified as purified forms of anthocyanins.

**Table 3:** Literature on the bioavailability of anthocyanins—human studies.

ACN Form	ACN Source	Treatment and Dose	Study Design	Participants	Bioaccessibility/Absorption Findings	Bioactivity Findings	Ref.
Capsule	Commercial (aronia berry)	500 mg	Single-dose pharmacokinetic trial	6 adults Age: 8–65 y BMI: 18.5–39 kg/m^2^ Former smokers	Plasma: 70–110% Urine: 43–119% Tmax: 1.0 h to 6.33 h	NA	[157]
Capsule	Commercial (bilberry, blackcurrant)	320 mg	Random, double-blind	58 adults Age: 56–67 y 24 wks	Plasma: 9.37 nmol/L	Antidiabetic, antioxidant, anti-dyslipidaemia	[132]
Juice	Commercial (20% blackcurrant)	250 mL of juice or control drink.	Randomised, cross-over, double-blind, placebo-controlled acute meal study.	9 males, 11 females; Age: 44.6 ± 13.3 y BMI: 23.9 ± 2.5 kg/m^2^	Plasma: ↑ plasma ascorbic acid, insulin, and urinary ACNs. Microbial metabolites were detected. Urine: (*p* < 0.001)	Vascular function: no effect	[136]
Juice	Commercial (blueberry, 216 mg cy)	250 mL daily for 28 days.	Randomised	4 males, 13 females; Age: 24–60 y	Urine: Total and parent anthocyanin varied 10-fold among all participants.	NA	[158]
Juice	Commercial Concord grape juice	200 mL of purple grape juice, control	Randomised, placebo-controlled, double-blind, counterbalanced-crossover study.	7 males, 13 females; Age: 18–35 y	NA	↑ Cognitive function	[142]
Juice	Red grape	400 mL red grape juice, organic red grape juice, and water (control).	Randomised, controlled, crossover study	5 males, 19 females; Age: 20–55 y BMI: 18–30 kg/m^2^.	NA	Antioxidant activity	[159]
Pulp, juice	Commercial (acai berry) Pulp, juice; 972 ± 27 mg/kg, 531 ± 0.2 mg/L ACN	7 mL/kg BW of acai pulp, clarified acai juice, and applesauce (negative control).	Acute four-way crossover	11 adults; Age: 21–31 y BMI: 17.8–25.9 kg/m^2^.	Plasma: Cmax 2321 and 1138 ng/L at tmax 2.2 and 2.0 h for pulp and juice Tmax 3 h for apple sauce, clarified acai juice, and acai pulp, and 2 h for the control beverage.	Antioxidant activity: in plasma pulp > juice. In urine, there is no difference.	[114]
Juice	Blood orange	600 mL juice; diet without juice for 21 days.	Crossover study	16 females Age: 20–27 y BMI: 16.0–23.3 kg/m^2^.	Plasma: ↑ plasma vitamin C, cyanidin-3-glucoside, â-cryptoxanthin, and zeaxanthin.	Oxidative stress: improved resistance of lymphocyte DNA to oxidative stress. whereas no effect was observed on the lipid oxidation biomarker.	[160]
Juice	Purple grumixama fruit	10 mL juice/kg BW.	Observational In vitro: breast cancer cells MDA-MB-231	10 females. Age: 29.3 ± 7.7 y BMI: 23 ± 3 kg/m^2^.	Urine: no significant difference.	Anticancer (breast)	[161]
Juice	Blueberry	250 mL juice (~216 mg C3G)		4 males, 13 females. Age: 24–60 y	Urine: 4% parent ACNs, and 96% ACN metabolites at 24 h. 226 known ACN and predicted ACN metabolites were identified, of which 91% were aglycones. AcnM persisted even after 5 days of abstaining from dietary Acn.		[162]
Juice	Blood orange	1 L of juice and control, 4 weeks.	Randomised, controlled, and crossover.	4 males, 4 females. Age: 23–44 y BMI: 18–27 kg/m^2^.	Plasma: Nil Urine: ↑ urinary excretion of ACN at 24 h	Cardioprotective: no effect	[163]
Concentrate	Montmorency tart cherry (MC)	30- or 60-mL	Randomised, double-blinded, and crossover.	12 males; Age: 26 ± 3 y BMI: 26.7 ± 3.2 kg/m^2^.	Plasma: ↑ ACN metabolites PCA, and VA at 1–2 h	Proliferation: no effect. Vascular function: ↑ only in combination with PCA and VA.	[57]
Wine and juice	Commercial Red wine, red grape juice; (279.6, 283.5 mg ACN)	400 mL of red wine or red grape juice	Non-randomised	4 males, 5 females; Age: 24–34 y BMI: 19.7–26.3 kg/m^2^.	Urine: <1% for both treatments after 7 h. Plasma: red wine > red grape juice (76.3% relative bioavailability)	NA	[164]
					Urine: TACNs are 0.18% in red wine and 0.23% in red grape juice.	Antioxidant activity: ↑ grape juice > wine	[111]
Smoothie, juice, and extract	Grape/blueberry (80:20)	0.33 litres of juice (841 mg ACN/L) or smoothie (983 mg ACN/L)	Randomised, double-blind, cross-over.	5 males, 5 females; Age: 23–27 y BMI: 19.6–25.1 kg/m^2^ in vitro: Caco-2	Plasma: Mal and peo > del, cy, pet juice > smoothie (80% relative bioavailability) Urine: juice > smoothie (71% relative bioavailability) Absorption: <0.1%.	NA	[89]
Puree	Strawberry	100 g, 200 g, and 400 g (~15 mmol, 30 mmol, and 60 mmol ACN)	Randomised, crossover	6 males, females; Age: 45 ± 8.4 y BMI: 24.4 ± 3.31 kg/m^2^.	Urine: 50% in the first 4 h. Pel-3g major ACN and are not saturated at dose ≤ 60μmol. Recovery increased linearly with increasing doses.		[165]
Fruit	Bilberry	180 g	Human	13 males Age: 22–24 y BMI: 18.3–22.8 kg/m^2^.	Plasma: AUC 0–6 h = 386.0 nmol h/mL; Cmax = 139.1 nM. Urine: 0.21%.	NA	[166]
Freeze-dried powder	Wild blueberries	25 g or placebo beverage.	A single-blind, randomised, two-arm crossover-controlled study	6 males, 6 females; Age: 20–45 y BMI: 25–33 kg/m^2^.	Plasma: ACNs were 1.1% and 3-CGA was 0.2%. Absorption: peonidin glycosides are the highest, and malvidin is the lowest.		[167]
Freeze-dried powder	Wild blueberry	240, 400, and 560 g and control drink.	Randomised controlled, double-blind, crossover.	21 males; Age: 18–40 y	Plasma: polyphenol metabolites (73% ± 2%) 32 total polyphenol metabolites were identified.	↑ Vascular function	[137]
Individually quick-frozen fruit	Blueberry	300 g (348 mg ACNs), control jelly	Randomised, crossover.	10 males; Age: 20.8 ± 1.6 y BMI: 22.5 ± 2.1 kg/m^2^	Plasma: ↑ at 1 and 2 h after consumption. No ACNs were detected after 24 h.	Antioxidant, Vascular function: no effect	[168]
Steamed	Red cabbage	100, 200, and 300 g	Randomised, crossover	6 males, 6 females; Mean age: 46 y Mean BMI: 25.4 kg/m^2^	Urine: 11 red cabbage ACNs and 4 CAN metabolites detected. Recovery: a linear decrease with increasing doses. Nonacylated anthocyanins 4 times higher than the acylated type.	NA	[169]
Cooked whole	Pigmented potatoes	150 g white (WP), yellow (YP), and purple (PP) potatoes, 6 wks.	Randomised, controlled, placebo, or crossover.	36 adults; Age: 18–40 y	NA	Anti-inflammatory: YP, PP Antioxidant activity: PP (160%)	[127]

Cy-3G: cyanidin-3-glucoside; PCA: protocatechuic acid; VA: vanillic acid; PC: polyphenolic content; ACN: anthocyanin; ACN-M: anthocyanin metabolites; TAC: total anthocyanin content; FMD: flow-mediated dilation; CRP: C-Reactive Protein; TBAR: thiobarbituric acid reactive substances; NADPH: nicotinamide adenine dinucleotide phosphate; CVD: cardiovascular disease; ↑: increased. Food or food products that have undergone single-stage extraction, such as solvent extraction, are classified as crude extracts, whereas those that have undergone more than three stages of extraction, including chromatographic technique, solid-phase extraction, and adsorption resins, are classified as purified forms of anthocyanins.

## 6. Conclusions

The growing body of research underscores the health benefits of anthocyanin consumption, leading to a surge in the availability of anthocyanin-rich extracts, or isolates, and encapsulated supplements. Despite extensive research on anthocyanin bioavailability, a critical gap exists in the literature, lacking simultaneous comparisons of anthocyanin bioavailability in whole foods versus isolates or extracts. As revealed in the current literature, the intricate interplay of anthocyanins with the food matrix has a profound impact on bioavailability, and therefore, this should not be overlooked when choosing between anthocyanin-rich whole foods and extracts. Further, it is difficult to compare the bioefficacies of anthocyanins from current individual studies due to variations in doses, extraction methods, methodologies, and the use of clinical models. This is also a limitation of this review, as it includes mostly individual studies due to the lack of parallel studies examining anthocyanin bioavailability when consumed as whole foods versus extracts. In addition, an in-depth evaluation of the role of biomarkers should be considered to understand the food matrix effect and mechanisms involved in the absorption of these compounds, which were not covered in this review.

Current findings, however, lean towards whole food consumption over purified or crude extracts of anthocyanins. Although there may not be any harm in consuming the purified form of phytonutrients such as anthocyanins as a dietary supplement, there may be less or no considerable benefit due to potentially missing out on synergistic interactions between anthocyanin compounds and the food matrix.

Direct comparative studies are imperative to decipher the nature of this inter-relationship between anthocyanins and food matrices—whether synergistic, neutral, or antagonistic. At the same time, it is important to consider the effects of processing techniques, encapsulation, and gut–microbiota interactions. Addressing this gap with robust study designs will not only benefit the food and nutraceutical industries but also empower health professionals to optimise the practical applications of anthocyanins, leveraging their therapeutic attributes. Moreover, such studies will eliminate interlaboratory differences and make the results more reliable and comparable. There is also a need to standardise the bioavailability study protocol with a study design that involves these stages: digestion, intestinal absorption, colonic fermentation, ability to exert a biological effect, and, if possible, a recommended standard unit of measurement.
